# Quality Control for Single Cell Analysis of High-plex Tissue Profiles using CyLinter

**DOI:** 10.1101/2023.11.01.565120

**Published:** 2023-11-01

**Authors:** Gregory J. Baker, Edward Novikov, Ziyuan Zhao, Tuulia Vallius, Janae A. Davis, Jia-Ren Lin, Jeremy L. Muhlich, Elizabeth A. Mittendorf, Sandro Santagata, Jennifer L. Guerriero, Peter K. Sorger

**Affiliations:** 1 Ludwig Center for Cancer Research at Harvard, Harvard Medical School, Boston, MA; 2 Laboratory of Systems Pharmacology, Program in Therapeutic Science, Harvard Medical School, Boston, MA; 3 Department of Systems Biology, Harvard Medical School, Boston, MA; 4 Harvard John A. Paulson School of Engineering and Applied Sciences, Harvard University, Cambridge, MA; 5 Systems, Synthetic, and Quantitative Biology Program, Harvard University, Cambridge, MA; 6 Breast Tumor Immunology Laboratory, Dana-Farber Cancer Institute, Boston, MA; 7 Breast Oncology Program, Dana-Farber/Brigham and Women's Cancer Center, Boston, MA; 8 Division of Breast Surgery, Department of Surgery, Brigham and Women's Hospital, Boston, MA; 9 Department of Pathology, Brigham and Women’s Hospital, Harvard Medical School, Boston, MA

**Keywords:** CyLinter, multiplex image analysis, quality control (QC), single-cell

## Abstract

Tumors are complex assemblies of cellular and acellular structures patterned on spatial scales from microns to centimeters. Study of these assemblies has advanced dramatically with the introduction of methods for highly multiplexed tissue imaging methods. These reveal the intensities and spatial distributions of 20-100 proteins in 10^3^–10^7^ cells per specimen in a preserved tissue microenvironment. Despite extensive work on extracting single-cell image data, all tissue images are afflicted by artifacts (e.g., folds, debris, antibody aggregates, optical effects, image processing errors) that arise from imperfections in specimen preparation, data acquisition, image assembly, and feature extraction. We show that artifacts dramatically impact single-cell data analysis, in extreme cases, preventing meaningful biological interpretation. We describe an interactive quality control software tool, CyLinter, that identifies and removes data associated with imaging artifacts. CyLinter greatly improves single-cell analysis, especially for archival specimens sectioned many years prior to data collection, including those from clinical trials.

## INTRODUCTION

Normal and tumor tissues are complex assemblies of many cell types whose proportions and properties are controlled by cell-intrinsic molecular programs and interactions among components of the tumor microenvironment (TME). For example, the initiation and progression of diseases such as cancer depend on competition between immunoediting by tissue resident or circulating immune cells, and immunosuppression by tumor cells. The relatively recent development of highly multiplexed tissue imaging methods (e.g., MxIF, CyCIF, CODEX, 4i, mIHC, MIBI, IBEX, and IMC)^1-7^ has made it possible to collect single-cell data on 20-100 proteins and other biomolecules in preserved 2D and 3D tissue microenvironments^4,8-11^. When these images are segmented and staining intensities are quantified, it is possible to generate single-cell data on cell types, their functional states, and their spatial interactions. Such data are powerful complements to that obtained from dissociative methods such as scRNA-seq^12-14^. Imaging approaches compatible with the formaldehyde-fixed, paraffin-embedded (FFPE) specimens universally acquired for clinical diagnosis are particularly powerful because they can tap into large archives of human biopsy and resection specimens^15,16^ and also assist in the study of mouse models of disease^17^.

Although machine learning methods operating at the pixel level can extract diagnostic features from histology images, particularly images of specimens stained with Hematoxylin and Eosin (H&E)—a common stain in clinical pathology^18^—most high-plex imaging studies aim to collect single-cell data that can be interpreted mechanistically. This requires segmenting images to identify individual cells and their features. The resulting single-cell data are recorded in “spatial feature tables”, which are analogous to count tables in scRNA-seq^19^. In their simplest form, spatial feature tables generated by pipelines such as MCMICRO (an automated multiplex image assembly and feature extraction pipeline)^19^ contain the X,Y coordinates of cells (commonly the centroids of the nuclei) and their integrated signal intensities^20^. Cell types (e.g., cytotoxic T cells immunoreactive to CD45, CD3 and CD8 antibodies) and their spatial locations are then inferred from these tables and spatial analysis performed to identify recurrent short- and long-range interactions significantly associated with an independent variable such as drug response, disease progression, or genetic perturbation.

High-plex imaging of human cohorts has been performed using both tissue microarrays (TMAs), which comprise 0.3 to 1.5 mm diameter “cores” from dozens to hundreds of clinical specimens arrayed on a slide, or by whole-slide imaging of single specimens up to 4 cm^2^. The latter is required for clinical research and diagnosis both to achieve sufficient statistical power^21^ and as an FDA requirement^22^. Analyzing imaging data from TMAs and whole-slide images requires specialized image processing algorithms (ideally organized into computational pipelines)^23^ because datasets can contain as many as 10^9^ cells. In this paper, we show that accurate processing of tissue images is complicated by the presence of imaging artifacts that contribute significant noise to single-cell data, confounding many types of image-derived, single-cell analysis. Tissue folds, slide debris (e.g., lint), and staining artifacts are commonly observed in histological specimens, especially those stored on glass slides for an extended period (e.g., archival samples stored for several years). Unfortunately, this is a common situation in the setting of clinical trial specimens. In our study, this scenario is represented by 25 slides from the TOPACIO clinical trial of Niraparib in Combination with Pembrolizumab in Patients with Triple-negative Breast Cancer or Ovarian Cancer (NCT02657889)^24^, which was completed in 2021. We demonstrate the impact of artifacts on data analysis by acquiring data from TOPACIO tissue specimens and by re-analyzing high-plex datasets from several recently published studies. We then develop an approach to removing single-cell data affected by microscopy artifacts using an interactive software tool (CyLinter, code and tutorial at https://labsyspharm.github.io/cylinter/) that is integrated into the Python-based Napari image viewer^25^. Finally, we show that CyLinter can salvage otherwise uninterpretable multiplex imaging data, from the TOPACIO trial for example. Our findings suggest that artifact removal should become a standard component of image processing pipelines used for spatial profiling of tissues.

## RESULTS

### Identifying recurrent image artifacts in multiplex IF images

**Extended Data Fig. 1 ∣:**
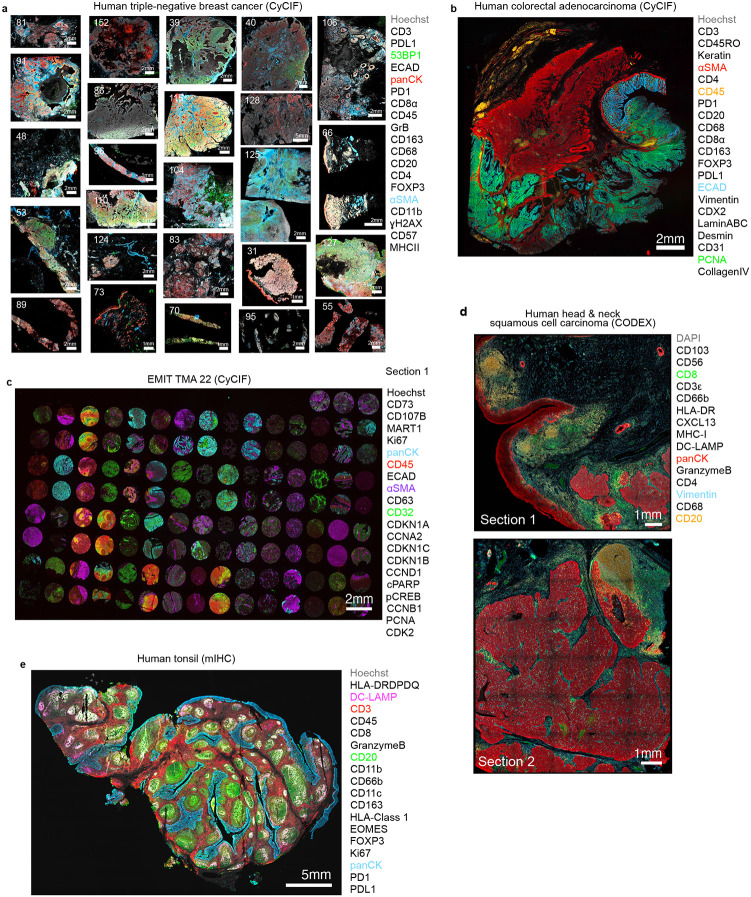
Overview of the five multiplex IF datasets used in this study. **a**, TOPACIO dataset (CyCIF): 25 human TNBC clinical specimens; numbers in upper left of each panel indicate sample number. Channels shown are Hoechst (gray, nuclei), 53BP1 (green, tumor), and pan-CK (red, tumor), and αSMA (blue, stroma). **b**, CRC dataset (CyCIF): a ~1.6 cm^2^ whole-slide section of primary human colorectal adenocarcinoma. Channels shown are Hoechst (gray, nuclei), αSMA (red, stroma), CD45 (orange, immune), ECAD (blue, epithelium), and PCNA (green, proliferating tumor). **c**, EMIT TMA22 dataset: 123 healthy and diseased human tissue cores ~2 mm in diameter arranged as a rectilinear grid on a single microscope slide. Channels shown are pan-CK (blue, epithelium), CD45 (red, immune), αSMA (purple, stroma), CD32 (green, immune), and CD45 (orange, immune). **d,** HNSCC dataset (CODEX): two ~1 cm^2^ whole-slide sections of human HNSCC. Channels shown are DAPI (gray, nuclei), CD8 (green, cytotoxic T cells), pan-CK (red, tumor), vimentin (blue, mesenchymal cells), and CD20 (orange, B cells). **e,** Normal human tonsil (mIHC): an ~3.2 cm x 1.9 cm whole-slide section of normal human tonsil. Channels shown are Hoechst (gray, nuclei), DC-LAMP (magenta, dendritic cells), CD3 (red, pan-T cells), CD20 (green, B cells), pan-CK (blue, epithelium). Column of markers to the right of each panel indicate the full marker set captured in those images. See Supplementary Table 1 for related sample identifiers and data access information.

To categorize imperfections and image artifacts commonly encountered in high-plex images of tissue, we examined five datasets collected using three different imaging methods: (1) 20-plex CyCIF^26^ images of 25 triple-negative breast cancer (TNBC) specimens, collected from patients in the TOPACIO clinical trial^24^; (2) a 22-plex CyCIF image of a colorectal cancer (CRC) resection^21^; (3) a 21-plex TMA dataset^23^ comprising 123 healthy and cancerous tissue cores; (4) 16-plex CODEX^27^ images of two sections from a single specimen of head and neck squamous cell carcinoma (HNSCC); and (5) a 19-plex mIHC^28^ image of normal human tonsil^23^ (Extended data Fig. 1a-e and Supplementary Table 1). CyCIF images were collected at 20x magnification (0.65μm/pixel) and processed using the MCMICRO^29^ image analysis pipeline to yield stitched and registered multi-tile image files and their associated single-cell spatial feature tables. Single-cell data were visualized as UMAP embedding following clustering with HDBSCAN—an algorithm for hierarchical density-based clustering^30^. Images were also inspected by experienced microscopists and board-certified histopathologists to identify imaging artifacts.

**Extended Data Fig. 2 ∣:**
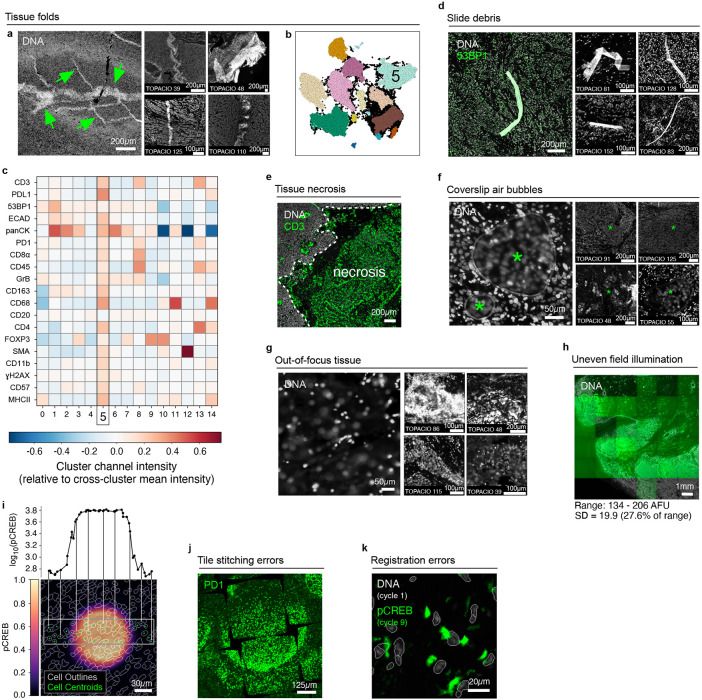
Recurring artifacts in whole slide immunofluorescence images of tissue and the effects on tissue-derived single-cell data. **a**, Tissue folding (green arrows) in normal human tonsil tissue imaged by mIHC as seen in the Hoechst channel (gray). Four additional examples of tissue folding in TOPACIO samples are shown at right. **b**, UMAP embedding shown in Fig. 1a colored by HDBSCAN clusters demonstrating the formation of a discrete cluster (cluster 5) caused by the tissue fold. **c**, Heatmap showing the difference between the channel signal intensities of individual clusters shown in Fig. 1a and Extended Data Fig. 2b and the average channel signal intensity across all clusters demonstrating that cluster 5 (corresponding to the tissue fold) exhibits greater-than-average intensities in all 20 channels in the image. **d**, Autofluorescent fiber seen in the 53BP1 (green) and Hoechst (gray) channels of TOPACIO sample 128. Four additional examples of slide debris in TOPACIO samples are shown at right. **e,** Necrosis in a region of tissue from TOPACIO sample 39 as seen in the CD3 (green) channel. **f**, Coverslip air bubbles (green asterisks) in TOPACIO sample 48 as seen in the Hoechst channel (gray). Four additional examples of coverslip air bubbles in TOPACIO samples are shown at right. **g**, Out-of-focus region of tissue in TOPACIO sample 55 as seen in the Hoechst channel (gray). Four additional examples of out-of-focus tissue in TOPACIO samples are shown at right. **h**, Uneven tile illumination in the HNSCC CODEX dataset as seen in the Cy5 channel. Hoechst (gray) shown to highlight tissue location. The standard deviation among per-tile median signal intensities was 19.93 arbitrary fluorescence units (AFU), 27.6% of the range (134-206 AFU). **i**, Illumination aberration in the pCREB (colormap) channel of EMIT TMA core 95 (dedifferentiated liposarcoma) with nuclear segmentation outlines (translucent contours) shown for reference. The line plot above shows the pCREB signal of cells highlighted by green scatter points in the image below. **j**, Tile stitching errors in the mIHC dataset of normal human tonsil as seen in the PD1 (green) channel. **k**, Cross-cycle image registration error in EMIT TMA core 64 (leiomyosarcoma) as demonstrated by superimposing the Hoechst (gray) signal from imaging cycle 1 and the pCREB (green) signal from imaging cycle 9.

**Fig. 1 ∣:**
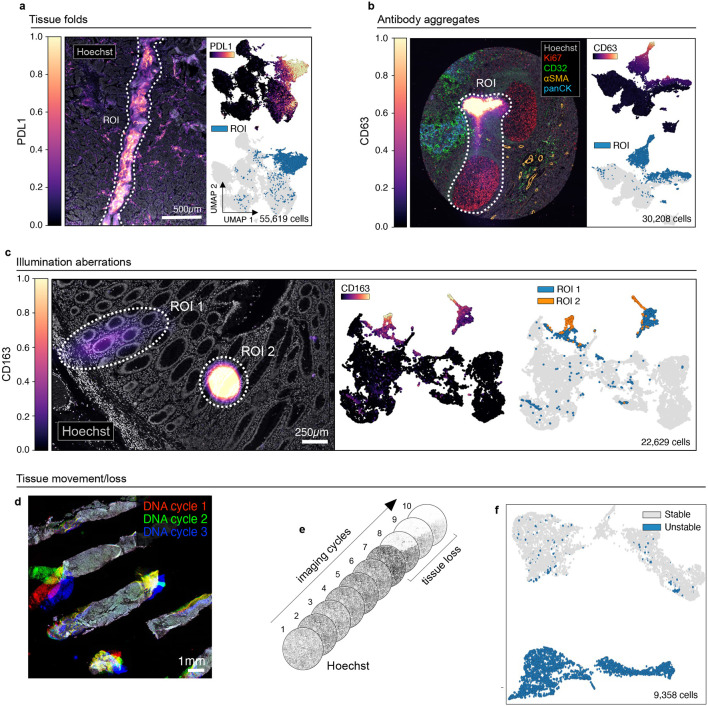
Recurring artifacts in whole slide immunofluorescence images of tissue and the effects on tissue-derived single-cell data. **a**, Left: Tissue folding ROI (dashed white outlines) in TOPACIO sample 110 as viewed in the PD-L1 (colormap) and Hoechst (gray) channels. Right: UMAP embeddings showing cells in the image colored by PD-L1 intensity (top) and whether they fall into the ROI shown at left (bottom). **b**, Left: Antibody aggregate in the CD63 (colormap) channel of EMIT TMA core 68 (human tonsil). Hoechst (gray), Ki67 (red), CD32 (green), αSMA (orange), and pan-CK (blue) are shown for context. Right: UMAP embedding showing cells from the image colored by CD63 intensity (top) and whether they fall within the ROI shown at left (bottom). **c,** Illumination aberrations in the CD163 (colormap) channel of the CRC dataset. Left: Image showing two bright spots (dashed white ROI outlines); Hoechst (gray) shown for reference. Right: UMAP embeddings showing 22-channel data from cells in the ROIs colored by CD163 intensity (left UMAP plot) and whether they are within one of the two ROIs shown in the corresponding image (right UMAP plot). **d**, Tissue detachment in TOPACIO sample 80 as demonstrated by superimposing Hoechst signals from three different CyCIF imaging cycles: 1 (red), 2 (green), 3 (blue). **e**, Progressive tissue loss in EMIT TMA core 1 (normal kidney cortex) across 10 imaging cycles as observed in the Hoechst channel (gray) where overt tissue loss is seen by cycle 8. **f**, UMAP embedding of cells from EMIT TMA core 1 (normal kidney cortex) colored by whether cells remained stable (gray data points) or became detached (blue data points) over the course of imaging demonstrating that unstable cells form discrete clusters in feature space.

All specimens comprised tissue sections cut at 5 μm thickness and mounted on slides in the standard manner. This involves sectionizing FFPE blocks with a microtome and floating sections on water prior to mounting on glass slides. Even in the hands of skilled histologists, this process can introduce folds in the tissue. We identified multiple instances of tissue folds in large and small specimens (such as TMA cores and core biopsies; Fig. 1a and Extended data Fig. 2a). Moreover, we found that cells within tissue folds corresponded to discrete clusters in UMAP feature space due to higher-than-average signal intensities across imaging channels relative to unaffected regions of tissue (Fig. 1a and Extended data Fig. 2b-c). Slide debris in the shape of lint fibers and hair were also common (Extended data Fig. 2d), as were bright antibody aggregates exhibiting non-specific staining patterns in the tissue that formed discrete clusters in UMAP space (Fig. 1b). Despite having relatively low numbers of segmented cells, regions of necrotic tissue also exhibited high levels of background antibody labeling (Extended data Fig. 2e). Some samples also contained air bubbles that were likely introduced when coverslips were overlayed on specimens prior to imaging (asterisks; Extended data Fig. 2f). Artifacts such as tissue folds and air bubbles can be reduced, but not completely eliminated, by skilled experimentalists.

Additional artifacts were incurred at the time of image acquisition. These included out-of-focus image tiles (in many cases due to sections not lying completely flat on the slide; Extended data Fig. 2g), fluctuations in background intensity between image tiles (Extended data Fig. 2h), and miscellaneous image aberrations that significantly increased signal intensities over image background and led to the formation of discrete clusters in UMAP space (Fig. 1c, Extended data Fig. 2i). We also observed errors in image tile alignment (Extended data Fig. 2j) and image registration (Extended data Fig. 2k), which represent image processing steps that are critical for deriving precise single-cell data. These stitching and registration errors can sometimes be solved computationally, although poor overall tissue quality and nuclear stain over-saturation can limit the accuracy of the recovered data.

Some artifacts are uniquely associated with cyclic imaging methods such as CyCIF^26,26,31^, CODEX^27^, and mIHC^28^ that generate high-plex images through repeated rounds of lower-plex imaging followed by fluorophore dissociation or inactivation. For instance, tissue movement (Fig. 1d) and progressive deterioration (Fig. 1e) over imaging cycles causes cells present in early rounds of imaging to be lost in later cycles. These cells appear negative for all subsequent markers, confounding cell type assignment and leading to artifactual clusters in feature space (Fig. 1f). The factors determining the extent of tissue loss from specimen to specimen remain incompletely understood, but we have found that tissue damage can occur from dewaxing or antigen retrieval^32^ and tissue sections with relatively low surface area (e.g., the fine-needle biopsies in TOPACIO samples 70, 89, 95, 96) or low cellularity (e.g., adipose tissue) are especially prone to tissue movement and cell loss.

In some cases, the origins of a given artifact were unknown, but based on our observations and published histology studies^33,34^, many likely arise from a combination of 1) pre-analytical variables (generally defined as variables arising prior to staining a specimen), 2) unwanted fluorescent objects introduced during staining, imaging, and washing steps (e.g., lint and antibody aggregates), 3) errors in data acquisition, and 4) the intrinsic properties of the tissue itself. Overall, we found that specimens from the TOPACIO dataset were the most severely affected by these artifacts, whereas the CRC specimen, from Lin et al.,^21^ which had been freshly-sectioned and carefully processed, was least affected. Only one slide was available from each TOPACIO patient due to high demand for specimens collected during the course of the clinical trial, making repeat imaging impossible. Thus, it was particularly important to correct imaging artifacts in the TOPACIO specimens.

### Microscopy artifacts obscure the analysis and interpretation of tissue-derived, single-cell data

**Extended Data Fig. 3 ∣:**
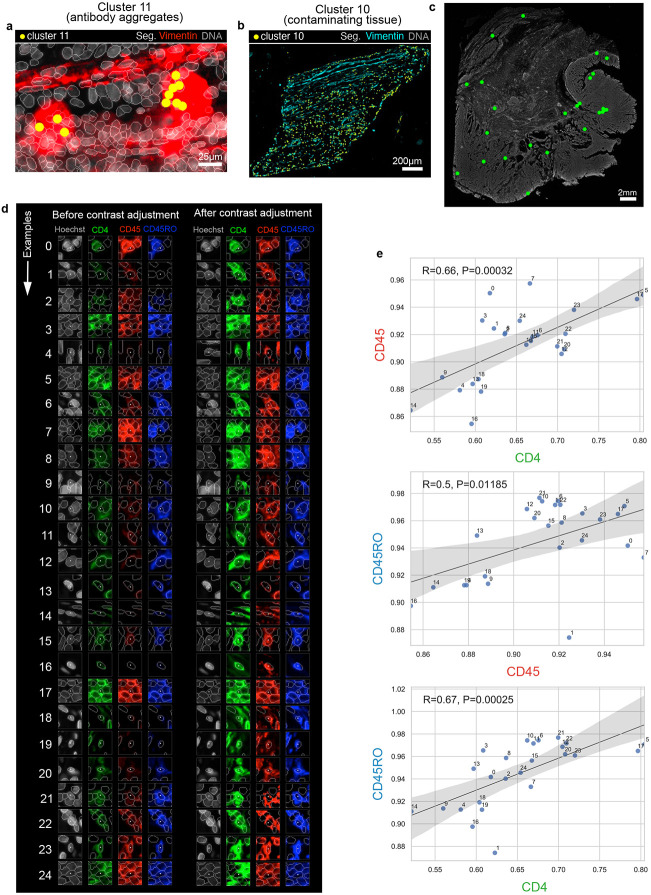
Evaluation of pre-QC cell clustering results from the CRC dataset. **a**, Anti-vimentin antibody aggregates (red) in a region of the CRC image. Yellow dots highlight cluster 11 cells that have formed due to this artefact; Hoechst (gray) shown for reference. **b**, Contaminating tissue at the top of the CRC image immunoreactive to anti-vimentin antibodies (cyan), which comprises CRC cluster 10 (yellow dots); Hoechst (gray) shown for reference. **c**, Location of cluster 3 cells (shown in Fig. 2k,m and Extended Data Fig. 3d) in the CRC image. **d**, Cluster 3 cells shown in Fig. 2k,m with channels shown separately for clarity: Hoechst (gray), CD4 (green), CD45 (red), and CD45RO (blue). Top rows are before contrast adjustment, bottom rows are post-contrast adjustment. **e**, Regression plots showing statistically significant correlation (two-sided, Pearson r, p < 0.05) among CD4, CD45, and CD45RO channels for cells shown in Fig. 2k,m and Extended Data Fig. 3d.

**Fig. 2 ∣:**
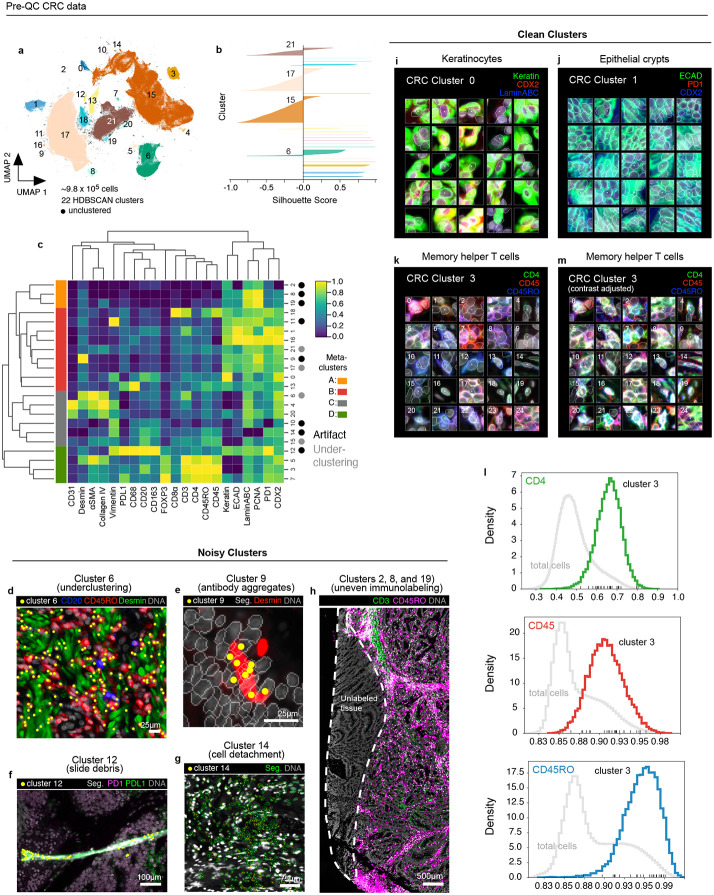
Evaluation of pre-QC cell clustering results from the CRC dataset. **a**, UMAP embedding of CRC data showing ~9.8 X 10^5^ cells colored by HDBSCAN cluster (numbered 0-21). Black scatter points represent unclustered (ambiguous) cells. **b**, Silhouette scores for CRC clusters shown in panel (a). Clusters 6, 15, 17, and 21 exhibit cells with negative silhouette scores indicative of under-clustering. **c**, CRC clustermap showing mean signal intensities of clustering cells normalized across clusters (row-wise). Four (4) meta-clusters defined by the clustermap dendrogram are highlighted. **d**, Cluster 6 cells (yellow dots) shown in a region of the CRC image demonstrating the co-clustering of distinct populations of B cells (CD20, blue), memory T cells (CD45RO, red), and stromal cells (desmin, green); Hoechst (gray) is shown for reference. **e,** Anti-desmin antibody aggregates (red) in a region of the CRC image. Yellow dots highlight cluster 9 cells which have formed due to this artefact; Hoechst (gray) shown for reference. **f**, Autofluorescent fiber in a region of the CRC image as seen in the PD1 (magenta) and PD-L1 (green) channels. Yellow dots highlight cluster 9 cells which have formed due to this artefact; Hoechst (gray) shown for reference. **g**, Cell detachment in a region of the CRC image as indicated by anucleate segmentation outlines (green). Yellow dots highlight cluster 14 cells which have formed due to this artefact; Hoechst (gray) shown for reference. **h**, Region of tissue at the bottom-left portion of the CRC image unexposed to anti-CD3e and anti-CD45RO antibodies used during imaging cycle 3 that led to the formation of CRC clusters 2, 8, and 19; Hoechst (gray) shown for reference. **i-k**, Top three most highly expressed markers (1: green, 2: red, 3: blue) for clusters 0 (keratinocytes, **i**), 1 (epithelial crypts, **j**), and 3 (memory helper T cells, **k**). A single white pixel at the center of each thumbnail highlights the reference cell. Nuclear segmentation outlines (translucent white outlines) and Hoechst (gray) are shown for reference. **l**, Density histograms of CD4 (green), CD45 (red), and CD45RO (blue) channels for cluster 3 cells superimposed on distributions of total cells as seen in the same channel (gray). Rugplots at the bottom of each histogram show where the 25 cluster 3 cells shown in panel (l) reside in each distribution. **m**, Cluster 3 cells shown in panel (l) after signal intensity cutoffs have been adjusted per image to improve their homogeneity of appearance.

Clustering with HDBSCAN yielded 22 clusters for the CRC dataset (~9.8x10^5^ cells total) with 0.7% of cells remaining unclustered due to ambiguous features (Fig. 2a). Silhouette analysis^35^ showed that four clusters (6, 15, 17, and 21) remained under-clustered (i.e., contained cells with negative silhouette scores) despite parameter tuning (Fig. 2b). Agglomerative hierarchical clustering based on mean marker intensities revealed four meta-clusters (Fig. 2c) that, upon initial inspection, appeared to correspond to tumor (meta-clusters A, B), stromal (C), and immune cells (D). However, multiple clusters contained cells with unexpected marker combinations. For example, cluster 6 cells contained cells from both immune and stromal lineages; inspection of the original image confirmed a mix of B cells, T cells, and stromal cells in this cluster (Fig. 2d). The formation of clusters 9 and 11 appeared to be caused by bright antibody aggregates in the desmin (Fig. 2e) and vimentin (Extended data Fig. 3a) channels, respectively, whereas contaminating lint fibers led to the formation of cluster 12 (Fig. 2f). Cell detachment was evident in cluster 14 (Fig. 2g), and cluster 10 comprised a domain of vimentin-positive tissue of unknown origin (Extended data Fig. 3b). Three additional artifactual clusters (2, 8, and 19; Fig. 2h) were caused by a region of tissue that was apparently not exposed to anti-CD3 and anti-CD45RO antibodies in imaging cycle 3. We reasoned that this artifact was likely due to human error during the performance of a complex 3D imaging study^21^.

To systematically inspect the individual cells comprising CRC clusters, we extracted and organized into image galleries 20 x 20 μm (30 x 30 pixel) image patches of randomly selected cells from each cluster (**Online Supplementary Fig. 1,** https://www.synapse.org/#!Synapse:syn24193163/files). To make the galleries easier to interpret, we limited the channels displayed to the three most highly expressed protein markers per cluster (based on a cluster-normalized heatmap; Fig. 2c). Clusters not overtly affected by artifacts (e.g., 0, 1, 3, 7, and 16) overwhelming contained cells with a consistent morphology and staining pattern. For example, CRC cluster 0 comprised a phenotypically homogenous group of keratinocytes (Fig. 2i), while CRC cluster 1 represented normal epithelial crypt cells with high-levels of E-Cadherin at intercellular junctions (Fig. 2j). Some clusters contained a single type of cell, but with remarkably non-uniform signal intensities. For example, cluster 3 cells expressed a combination of markers consistent with memory helper T cells (i.e. CD45, CD4, and CD45RO) but with high variation among replicate thumbnails (Fig. 2k,l). These cells were drawn from multiple tissue regions, not a single region affected by artifacts (Extended data Fig. 3c) and manual adjustment of image contrast on a per-channel and per-cell basis made the cells appear more uniform (Fig. 2m and Extended data Fig. 3d). As expected for this T cell subset, CD45, CD4, and CD45RO staining was significantly correlated in individual cells (R=0.5 to 0.67; Extended data Fig. 3e), explaining the tight cluster in the UMAP embedding, but with sufficient variation that made multi-channel images of the same cell type look quite different. We believe that this reflects natural biological variation—not simply dataset noise.

**Extended Data Fig. 4 ∣:**
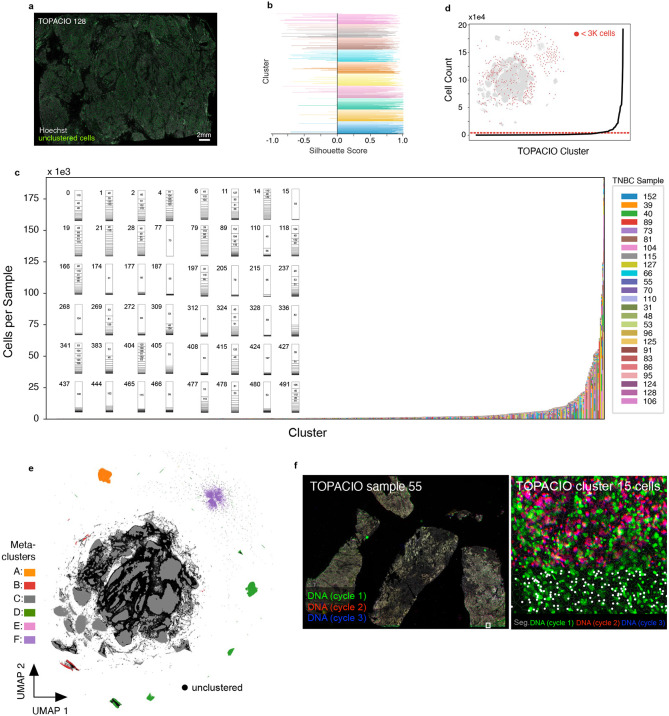
Evaluation of pre-QC cell clustering results from the TOPACIO dataset. **a**, Spatial distribution of unclustered cells (green dots) from the pre-QC TOPACIO embedding (Fig. 3a) as shown in sample 55 demonstrating no discernable pattern of sampling bias; Hoechst (gray) shown for reference. **b**, Silhouette scores for pre-QC TOPACIO clusters shown in (Fig. 3a). **c**, Stacked bar charts showing the relative contribution of each patient sample to each cluster. Inset shows more detailed views for 48 of the 492 clusters that were selected at random for curating image patch galleries for visual inspection of cells comprising these clusters (**Online Supplementary Fig. 2**). **d**, Line plot showing cell counts per TOPACIO cluster. Clusters with cell counts below the horizonal dashed red line are those with fewer than 3K cells which are highlighted in the TOPACIO embedding (inset) by red scatter points at their relative positions. **e**, TOPACIO embedding colored by meta-clusters shown in Fig. 3b. **f**, TOPACIO sample 55 at low (left) and high (right) magnification showing Hoechst signals for the first three imaging cycles: 1 (green), 2 (red), and, 3 (blue) superimposed to demonstrate cross-cycle image alignment errors in this sample. Small white box at the bottom-right of the low magnification image shows the location of the higher magnification image. White dots in the image magnification image highlight cluster 15 cells which have formed due to this image alignment artefact.

**Fig. 3 ∣:**
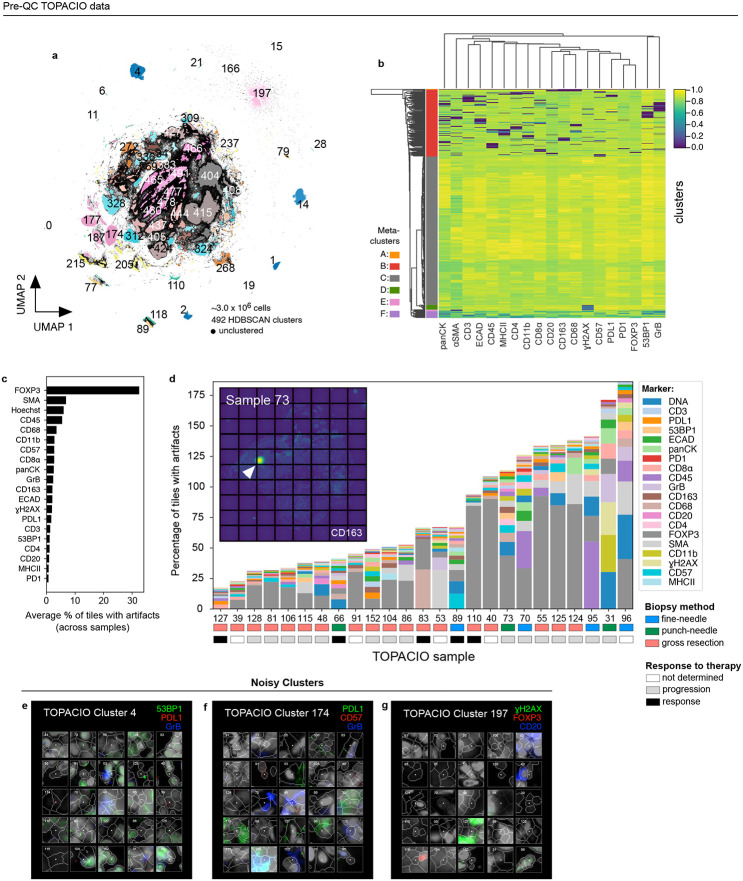
Evaluation of pre-QC cell clustering results from the TOPACIO dataset. **a**, UMAP embedding of TOPACIO data showing ~3 X 10^6^ cells colored by HDBSCAN cluster. Black scatter points represent unclustered (ambiguous) cells. **b**, TOPACIO clustermap showing mean signal intensities of clustering cells normalized across clusters (row-wise). Six (6) meta-clusters defined by the clustermap dendrogram at the left are highlighted. **c**, Bar chart showing the average percentage of channel-specific tiles affected by a visual artifact across the 25 TOPACIO samples. **c**, Stacked bar chart showing the percentage of channel-specific image tiles per TOPACIO sample affected by miscellaneous visual artifacts. Inset shows an example illumination aberration in the CD163 channel of sample 73. Categories for tissue biopsy method and patient treatment response are indicated below each bar. **e-g**, Top three most highly expressed markers (1: green, 2: red, 3: blue) for clusters 4 (**e**), 174 (**f**), and 197 (**g**) which are all severely affected by dataset noise. A single white pixel at the center of each image highlights the reference cell. Nuclear segmentation outlines (translucent white outlines) and Hoechst (gray) are shown for reference.

The 20-plex TOPACIO dataset gave rise to 492 HDBSCAN clusters (among a subset of ~3.0 x 10^6^ cells drawn from the ~1.9 x 10^7^ total segmented nuclei), with 875,204 (29%) of cells remaining unclustered and exhibiting no discernable spatial pattern (Fig. 3a and Extended data Fig. 4a). Most clusters were associated with positive silhouette scores, indicating a good fit (Extended data Fig. 4b). While some clusters arose from cells in a single specimen, the majority (441/492) contained cells from more than half of the 25 TOPACIO samples (Extended data Fig. 4c). This included many small clusters containing fewer than 3,000 cells (392/492, Extended data Fig. 4d). Agglomerative hierarchical clustering based on mean marker intensities revealed six meta-clusters (Fig. 3b). However, the heatmap exhibited an unexpected pattern of dichotomous marker expression with very bright signals for some markers and very dim signals for others. The exception was meta-cluster C, comprising the majority of cells (~1.7 x10^6^), which exhibited bright signals across all channels (Fig. 3b) and was located towards the center of the UMAP embedding (Extended data Fig. 4e). On further inspection, we found that a significant image alignment problem at the bottom of patient sample 55 gave rise to the single clustering (15) comprising meta-cluster A (Extended data Fig. 4f). Meta-clusters B, D, E, and F found to be caused by the presence of cells with channel intensities at or near zero resulting from image background subtraction (see Supplementary Note 1). Overall, the same type of analysis that generated interpretable data for the CRC specimen, albeit with some artifacts, yielded nearly unintelligible results with the TOPACIO dataset.

Visual inspection of the 156,300 individual channel image tiles comprising the TOPACIO dataset in down-sampled images revealed that ~5,487 tiles (3.5%) were affected by either antibody aggregates, illumination aberrations, or slide debris, with FOXP3 being the most affected channel in which artifacts were present in >30% of tiles across all samples (Fig. 3c), which was likely due to insufficient antibody washing prior to imaging. Artifacts tended to be less abundant in gross tissue resections compared to fine-needle and punch-needle biopsies, but were uncorrelated with patient response to therapy, as expected (Fig. 3d). Image patch galleries drawn at random from 48 of the 492 TOPACIO clusters also revealed numerous tissue and imaging artifacts including bright fluorescent signals, over-saturated nuclear counterstain, poor segmentation, and low fluorescent signals (Fig. 3e-g and **Online Supplementary Fig. 2**). Thus, the number of artifacts in the TOPACIO specimens was substantially higher than in the CRC specimen and we speculated that this might explain the uninterpretability of much of the data.

### Identifying and removing noisy single-cell data with CyLinter

**Extended Data Fig. 5 ∣:**
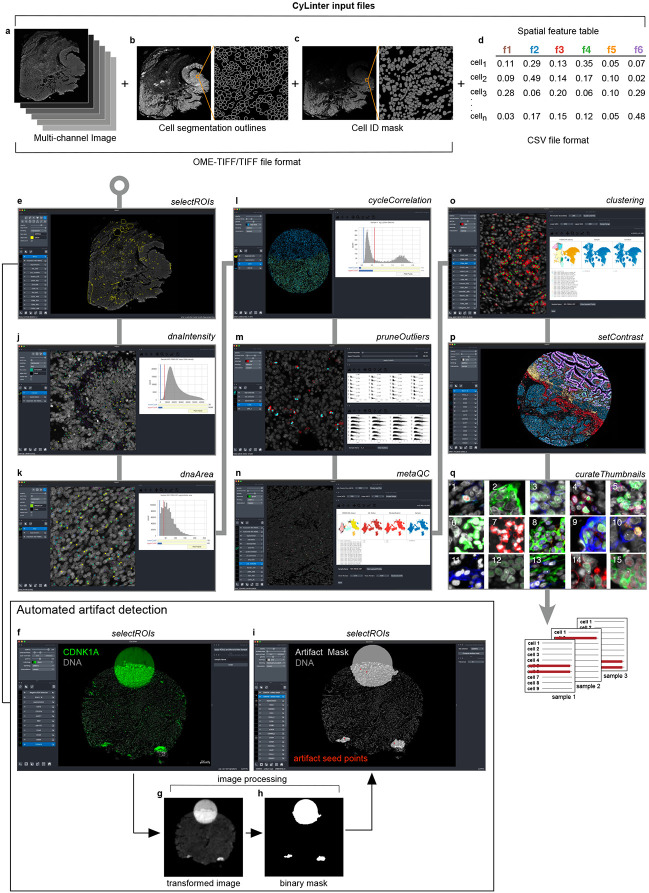
Identifying and removing noisy single-cell data points with CyLinter. **a-d:** CyLinter input: **a**, Multiplex microscopy file, **b**, Cell segmentation outlines, **c**, Cell ID mask, **d**, Single-cell feature table. **e**, ROI selection module: multi-channel images are viewed to identify and gate on regions of tissue affected by microscopy artifacts (in the default negative selection mode). **f-i**, Demonstration of automated artifact detection in CyLinter. **f**, CyLinter’s *selectROIs* module showing artifacts in the CDKN1A (green) channel of EMIT TMA core 18 (mesothelioma). **g**, Transformed version of the original CDKN1A image such that artifacts appear as large, bright regions relative to channel intensity variations associated with true signal of immunoreactive cells which are suppressed. **h**, Local intensity maxima are identified in the transformed image and a flood fill algorithm is used to create a pixel-level binary mask indicating regions of tissue affected by artifacts. In this example, the method identifies three artifacts in the image: one fluorescence aberration at the top of the core, and two tissue folds at the bottom of the core. **i**, CyLinter’s *selectROIs* module showing the binary artifact mask (translucent gray shapes) and their corresponding local maxima (red dots) defining each of the three artifacts. **j**, DNA intensity filter: histogram sliders are used to define lower and upper bounds on nuclear counterstain single intensity. Cells between cutoffs are visualized as scatter points at their spatial coordinates in the corresponding tissue for gate confirmation or refinement. **k**, Cell segmentation area filter: histogram sliders are used to define lower and upper bounds on cell segmentation area (pixel counts). Cells between cutoffs are visualized as scatter points at their spatial coordinates in the corresponding tissue for gate confirmation or refinement. **l**, Cross-cycle correlation filter: applicable to multi-cycle experiments. Histogram sliders are used to define lower and upper bounds on the log-transformed ratio of DNA signals between the first and last imaging cycles. Cells between cutoffs are visualized as scatter points at their spatial coordinates in their corresponding tissues for gate confirmation or refinement. **m**, Channel outlier filter: the distribution of cells according to antibody signal intensity is viewed for all sample as a facet grid of scatter plots (or hexbin plots) against cell area (y-axes). Lower and upper percentile cutoffs are applied to remove outliers. Outliers are visualized as scatter points at their spatial coordinates in their corresponding tissues for gate confirmation or refinement. **n**, MetaQC module: unsupervised clustering methods (UMAP or TSNE followed by HDBSCAN clustering) are used to correct for gating bias in prior data filtration modules by thresholding on the percent of each cluster composed of clean (maintained) or noisy (redacted) cells. **o**, Unsupervised cluster methods (UMAP or TSNE followed by HDBSCAN) are used to identify unique cell states in a given cohort of tissues. **p**, Image contrast adjustment: channel contrast settings are optimized for visualization on reference tissue which are applied to all tissues in the cohort. **q**, Evaluate cluster membership: cluster quality is checked by visualizing galleries of example cells drawn at random from each cluster identified in the *clustering* module (Extended Data Fig. 5o).

**Fig. 4 ∣:**
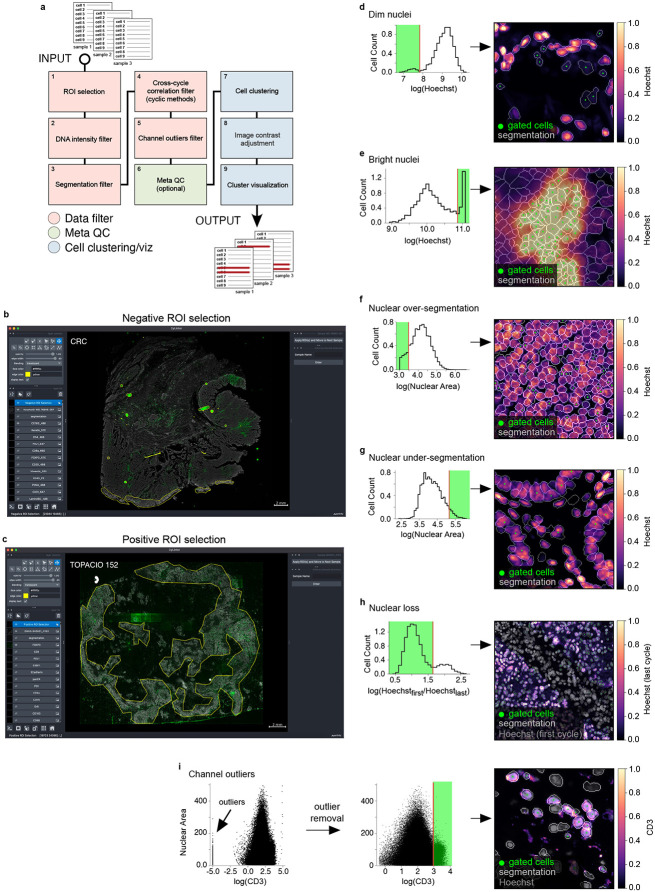
Identifying and removing noisy single-cell data points with CyLinter. **a**, Schematic representation of the CyLinter workflow. Modules are colored by type: data filtration (red), metaQC (green), and cell clustering/visualization (blue). **b**, CyLinter’s ROI selection module showing the CRC image with ROIs (yellow outlines) applied to various artifacts in the CD163 channel demonstrating negative ROI selection (i.e., cells in ROIs are dropped from further analysis). **c**, CyLinter’s ROI selection module showing TOPACIO sample 152 with ROIs (yellow outlines) applied to regions devoid of artifacts in the FOXP3 channel demonstrating positive ROI selection (i.e., cells in ROIs are retained for further analysis). **d**, Left: Density histogram of mean Hoechst signal for cells in EMIT TMA core 12 (non-neoplastic lung). Right: Hoechst (colormap) in a region of the same core demonstrating dim nuclei (green dots) falling to the left of the red gate in the corresponding histogram. Nuclear segmentation outlines are shown for reference (translucent outlines). **e**, Left: Density histogram of mean Hoechst signal for TOPACIO sample 110. Right: Hoechst (colormap) in a region of the same sample demonstrating bright nuclei (green dots) caused by tissue bunching that falls to the right of the gate in the corresponding histogram. Nuclear segmentation outlines are shown for reference (translucent outlines). **f**, Left: Density histogram of mean Hoechst signal for the CRC sample. Right: Hoechst (colormap) in a region of the sample demonstrating over-segmented cells (green dots) falling to the left of the red gate in the corresponding histogram. Nuclear segmentation outlines are shown for reference (translucent outlines). **g**, Left: Density histogram of mean Hoechst signal for EMIT TMA core 84 (non-neoplastic colon). Right: Hoechst (colormap) in a region of the sample demonstrating under-segmented cells (green dots) falling to the right of the red gate in the corresponding histogram. Nuclear segmentation outlines are shown for reference (translucent outlines). **h**, Left: Density histogram of the log(ratio) between Hoechst signals from the first and last CyCIF imaging cycles for EMIT TMA core 74 (renal cell carcinoma). Right: Hoechst (last cycle, colormap) superimposed on Hoechst (first cycle, gray) in a region of the sample demonstrating the selection of stable cells (green dots) falling to the left of the red gate in the corresponding histogram. Nuclear segmentation outlines are shown for reference (translucent outlines). **i**, Scatter plot showing CD3 (x-axis) vs. nuclear segmentation area (y-axis) of cells from TOPACIO sample 152 before (left) and after (right) outlier removal and signal rescaling (0-1). Image at right shows CD3 (colormap) and Hoechst (gray) signals in a region of the same sample with CD3^+^ cells (green dots) falling to the right of the red gate in the scatter plot in which outliers have been removed. Nuclear segmentation outlines are shown for reference (translucent outlines).

To remove imaging artifacts from tissue images we developed the CyLinter plugin for the Napari^25^ multi-channel image viewer. The tool consists of a set of Python-based QC modules that process single-cell data for identification and removal of image artifacts using computer-assisted human review (Fig. 4a) and can be incorporated into the MCMICRO image processing pipeline^29^. CyLinter takes four files as input for each specimen: 1) a stitched and registered multiplex image (TIFF/OME-TIFF format), 2) a single-channel binary image showing the boundaries between segmented cells, 3) a cell segmentation mask generated by MCMICRO or a stand-alone segmentation algorithm, and 4) a spatial feature table (CSV format)^20^ comprising the location and computed signal intensities for each segmented cell within an image derived from the segmentation mask (Extended data Fig. 5a-d, respectively). With a dataset comprising multiple images and spatial feature tables, CyLinter aggregates single-cell data into one Pandas (Python) dataframe^36^. During QC, cells affected by artifacts are then removed from the dataframe. CyLinter is flexible, as it allows QC modules to be run iteratively and progress to be bookmarked within and between modules.

The first CyLinter module, *selectROIs*, is used to view a multi-channel image to identify obvious artifacts, such as regions of tissue damage, antibody aggregates, and large illumination aberrations (Extended data Fig. 5e). Lasso tools native to the Napari image viewer are used to define regions of interest (ROIs) corresponding to artifacts and affected cells are removed from the spatial feature table (see https://labsyspharm.github.io/cylinter/ for details). We found that negative selection (in which highlighted cells are dropped from further analysis) worked effectively for the CRC image (Fig. 4b), but the TOPACIO dataset was affected by too many artifacts to use such an approach. Thus, CyLinter implements an optional positive ROI selection mode, in which users select regions of tissue devoid of artifacts for retention in the dataset (Fig. 4c). Although human identification of artifacts is effective, it can be slow. Therefore, CyLinter includes a companion algorithm that works with the *selectROIs* module to automatically flag likely artifacts for human review (Extended data Fig. 5f-i). The algorithm is based on classical image processing approaches (see Methods) that identify features with intensities lying outside the distribution of biological signals (e.g., illumination aberrations, antibody aggregates, and tissue folds). More sophisticated machine learning models such as multi-layer perceptrons and other neural networks are now being developed for integration into the CyLinter QC pipeline^37,38,39^.

CyLinter’s *dnaIntensity* module allows users to inspect histogram distributions of per-cell mean nuclear intensities within each cycle. Nuclei at the extreme left side of the distribution often correspond to cells lying outside of the focal plane (Fig. 4d), while those to the right correspond to poorly segmented cells and those within tissue folds (Fig. 4e); this module redacts data based on user assigned lower and upper thresholds (Extended data Fig. 5j). Instances of over and under-segmentation can be identified based on the area of each segmentation instance (typically expressed in number of pixels) followed by their removal using the *dnaArea* module (Extended data Fig. 5k). This method was particularly effective at removing many over-segmented cells in the CRC image (Fig. 4f) and under-segmented cells which were common among tightly-packed columnar epithelial cells in normal colon specimens (e.g., EMIT TMA core 84; Fig. 4g).

In cyclic imaging methods, nuclei are re-imaged every cycle^40,41^ and CyLinter’s *cycleCorrelation* module exploits this to identify cells that were lost or substantially damaged during imaging by computing histograms of log_10_-transformed ratios of DNA intensity between the first and last imaging cycles for a particular image (Extended data Fig. 5l). Cells that are lost give rise to a discrete peak with log_10_[DNA_1_/DNA_n_] > 0 and gating the histogram eliminates these cells from the data table (Fig. 4h). A further module (*pruneOutliers*) exploits the fact that artifacts are often (but not always) associated with brighter signals relative to real ones. With the *pruneOutliers* module, it is possible to simultaneously visualize histograms of per-cell signal intensities from all samples in a tissue batch, then assign lower and upper percentile cutoffs (Fig. 4i and Extended data Fig. 5m). Not all channels will contain artifacts, and cells falling outside of the thresholds can therefore be visualized in tissues to ensure that selected data points are indeed artifacts. We have found that this approach is particularly effective at removing cells affected by antibody aggregates which can be small, and tedious to curate through the *selectROIs* module.

### Correcting for bias in user-guided histology QC via unsupervised cell clustering

Human-guided artifact detection is subject to errors and biases. CyLinter therefore implements a *metaQC* module (Extended data Fig. 5n) that performs unsupervised clustering on equal combinations of redacted and retained data. Cells flagged for redaction that fall within predominantly clean clusters in the retained data can be added back to the dataset, while those retained in the dataset that co-cluster with predominantly noisy cells (which were presumably missed during QC) can be removed from the data table. Like the *metaQC* module, CyLinter’s *clustering* module (Extended data Fig. 5o) allows users to perform UMAP^42^ or t-SNE^43^ data dimensionality reduction and HDBSCAN^30^ density-based clustering to identify discrete cell populations in high-dimensional feature space. After achieving an optimal cluster solution, the *setContrast* module adjusts per-channel image contrast settings (Extended data Fig. 5p) that are applied to all tissues in a batch. The *curateThumbnails* module then selects individual cells at random from each cluster and curates image galleries for visual confirmation of clusters as *bona fide* cell states or residual dataset noise (Extended data Fig. 5q). Together, these additional clustering-based QC steps allow a user to revise any prior cleaning and clustering modules using a more objective approach than image inspection alone.

### Impact of CyLinter-based quality control on the TOPACIO and CRC datasets

**Extended Data Fig. 6 ∣:**
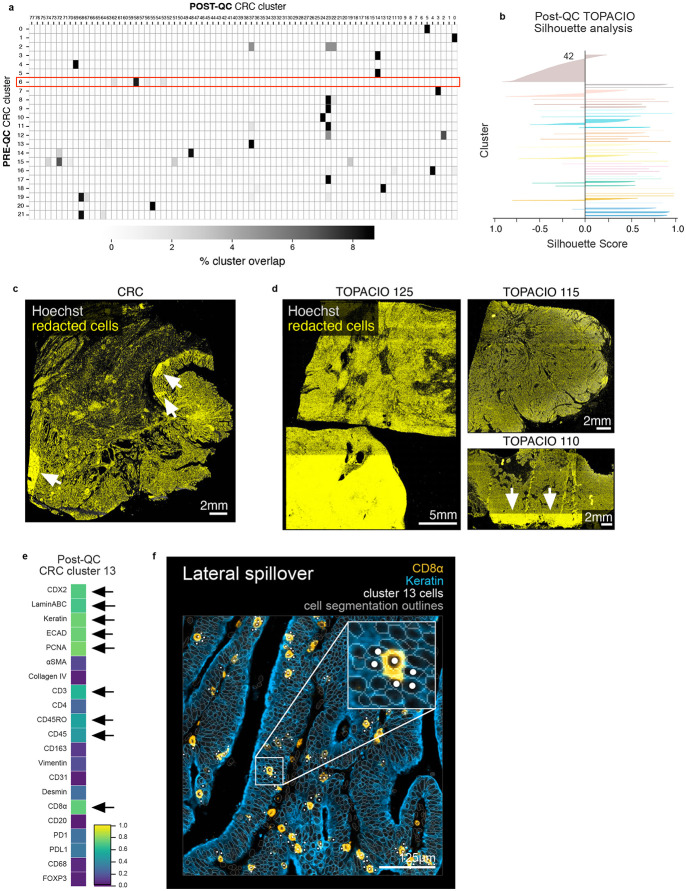
Overlap between pre- and post-QC CRC clusters, TOPACIO silhouette scores, tissue distribution of redacted cells, and demonstration of lateral spillover in post-QC CRC data. **a**, Percent overlap between pre-QC CRC clusters (rows) and post-QC CRC clusters (columns) showing that several pre-QC clusters are in fact made up of multiple post-QC clusters (i.e., different cell types). **b**, Silhouette scores for post-QC TOPACIO clusters shown in Fig. 6b. Cluster 42 is an under-clustered population. **c**, Cells redacted by CyLinter from the CRC dataset demonstrating no discernable pattern (or bias) in the removal of cells from the image with the exception of select ROIs (highlighted by white arrows) used to remove focal artifacts. **d**, Cells redacted by CyLinter from three arbitrary samples from the TOPACIO dataset demonstrating no discernable pattern (or bias) in the removal of cells from the images with the exception of select ROIs (highlighted by white arrows) used to remove focal artifacts. **e**, Mean signal intensities for post-QC CRC cluster 13 cells. Black arrows point to bright channels. **f**, Post-QC CRC cluster 13 cells (white dots) shown in the context of the CRC image demonstrating later spillover between keratin+ tumor cells (blue) and CD8α^+^ T cells (orange). Nuclear segmentation outlines (translucent outlines) are shown for reference.

**Fig. 5 ∣:**
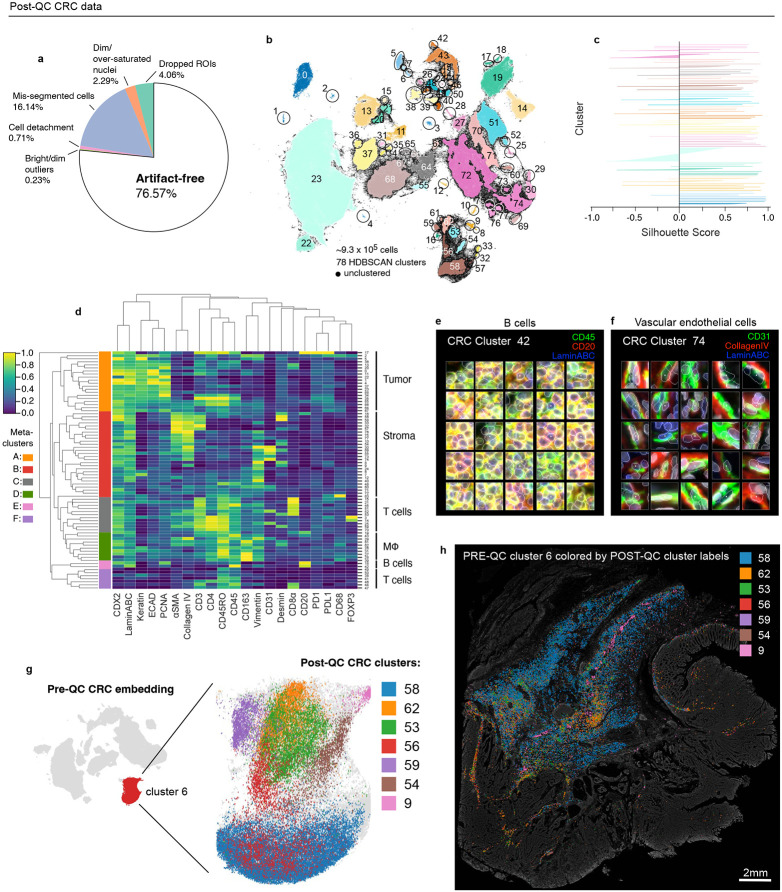
Cleaning the CA Dataset with CyLinter. **a**, Fraction of cells in the CRC dataset redacted by each QC filter in the CyLinter pipeline. Dropped ROIs (cells dropped using the *selectROIs* module), Dim/over-saturated nuclei (cells dropped using the *dnaIntensity* module), mis-segmented cells (cells dropped using the *areaFilter* module), cell detachment (cells dropped using the *cycleCorrelation* module), bright/dim outliers (cells dropped used the *pruneOutliers* module), artifact-free (cells remaining after QC). **b**, UMAP embedding of CRC data showing 933,253 cells colored by HDBSCAN cluster. Black scatter points represent unclustered (ambiguous) cells. **c**, Silhouette scores for CRC clusters shown in Fig. 5b. **d**, CRC clustermap showing mean signal intensities of clustering cells normalized across clusters (row-wise). Six (6) meta-clusters defined by the clustermap dendrogram at the left are highlighted. **e-f**, Top three most highly expressed markers (1: green, 2: red, 3: blue) for clusters 42 (B cells, **e**) and 74 (vascular endothelial cells, **f**). A single white pixel at the center of each image highlights the reference cell. Nuclear segmentation outlines (translucent outlines) and Hoechst (gray) are shown for reference. **g**, Pre-QC CRC embedding showing the position of cluster 6 (red, inset) and its composition according to post-QC CRC clusters. **h**, Locations of cells in pre-QC cluster 6 colored by their post-QC cluster number (Fig. 5b) showing that pre-QC cluster 6 is composed of cells occupying distinct regions with the muscularis propria of the CRC image—a non-cancerous, smooth muscle-rich region of the tissue.

Applying CyLinter to the CRC dataset resulted in the removal of ~23% of total cells (Fig. 5a). Over-segmentation was the largest problem, affecting ~16% of cells, with 2% or less of the data being dropped by any of one of the remaining QC modules. Thus, use of better segmentation algorithms (many of which are in development) would in principle have allowed 93% of the data to be retained. Using CyLinter to perform HDBSCAN clustering on the cleaned CRC dataset, we identified 78 clusters (Fig. 5b)—56 more than the pre-QC CRC clustering (Fig. 2a). Silhouette scores were predominantly positive, suggesting an optimal clustering (Fig. 5c) and agglomerative hierarchical clustering yielded six meta-clusters with marker expression patterns corresponding to populations of tumor cells (meta-cluster A; Fig. 5d), stromal cells (B), memory T cells (C), macrophages (D), B cells (E), and effector T cells (F). Using *curateThumbnails* we confirmed that all 78 clusters were free of visual artifacts (Fig. 5e,f and **Online Supplementary Fig. 3**) and reasoned that the increase in the number of clusters in the post-QC CRC embedding was due to the removal of pre-QC outliers that constrained the remainder of the cells to a relatively narrow region of the UMAP feature space. For example, pre-QC CRC cluster 6 (Fig. 2d) resolved in the post-QC embedding into seven discrete sets of cells with distinct markers and spatial locations (Fig. 5g,h, Extended data Fig. 6a and **Online Supplementary Fig. 3**). We concluded that even small numbers of artifacts corresponding to outliers in image intensity dramatically affect data interpretation.

**Fig. 6 ∣:**
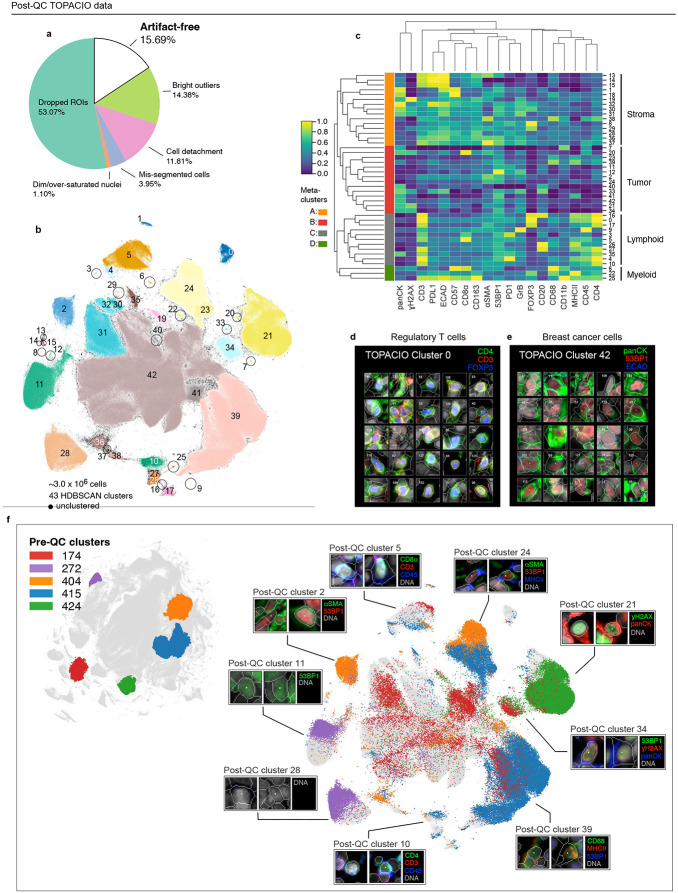
Cleaning the TOPACIO Dataset with CyLinter. **a**, Fraction of cells in the TOPACIO dataset redacted by each QC filter in the CyLinter pipeline. Dropped ROIs (cells dropped using the *selectROIs* module), Dim/over-saturated nuclei (cells dropped using the dnaIntensity module), mis-segmented cells (cells dropped using the *areaFilter* module), cell detachment (cells dropped using the *cycleCorrelation* module), bright/dim outliers (cells dropped used the *pruneOutliers* module), artifact-free (cells remaining after QC). **b**, UMAP embedding of TOPACIO data showing 2,970,240 cells colored by HDBSCAN cluster. Black scatter points represent unclustered (ambiguous) cells. **c**, TOPACIO clustermap showing mean signal intensities of clustering cells normalized across clusters (row-wise). Six (4) meta-clusters defined by the clustermap dendrogram at the left are highlighted. **d-e**, Top three most highly expressed markers (1: green, 2: red, 3: blue) for clusters 0 (regulatory T cells, **d**) and 42 (breast cancer cells, **e**). A single white pixel at the center of each image highlights the reference cell. Nuclear segmentation outlines (translucent outlines) and Hoechst (gray) are shown for reference. **f**, Left: Pre-QC TOPACIO UMAP embedding showing the location of five clusters selected at random. Right: location of those five random pre-QC clusters in the post-QC and their corresponding thumbnails, indicating multiple types of biologically extant cell states in the post-QC embedding.

In the case of the TOPACIO dataset, CyLinter removed 84% of the total cells. The majority (~53%) were removed during the process of positive ROI selection, in which selected regions of tissue largely devoid of obvious artifacts were curated (Fig. 6a). Bright outliers attributed to antibody aggregates, cell detachment, mis-segmentation, and dim/over-saturated nuclei accounted for ~14%, 12%, 4%, and 1% of redacted data, respectively. Overall, the post-QC TOPACIO dataset comprised ~3.0 x 10^6^ cells (~16% of total segmentation instances in the pre-QC dataset) and HDBSCAN clustering identified 43 clusters in the UMAP embedding (Fig. 6b). Silhouette analysis revealed positive scores for cells in most clusters except for those in cluster 42 (Extended data Fig. 6b) – the largest cluster in the embedding. Agglomerative hierarchical clustering based on mean marker intensities yielded four meta-clusters corresponding to stromal (meta-cluster A; Fig. 6c), tumor (B), lymphoid (C), and myeloid (D) cells. Using CyLinter’s *curateThumbnails* module to inspect image patches of cells from each cluster, we found that most cells had a high degree of concordance in morphology and marker expression among replicates and were consistent with extant cell types (**Online Supplementary Fig.4**). For example, post-QC TOPACIO cluster 0 corresponded to cells with small, round, nuclei with intense plasma membrane staining for CD4 and nuclear staining for FOXP3 (Fig. 6d), consistent with regulatory T cells; while cells in cluster 42 had high panCK and moderate ECAD staining (the latter at cell-cell junctions) indicative of de-differentiating breast epithelial cells (Fig. 6e). Coloring the post-QC UMAP embedding by select pre-QC cluster labels confirmed that many pre-QC clusters were in fact composed of different cell types (Fig. 6f). For example, pre-QC TOPACIO cluster 174 contained cells that resolved into at least 14 different post-QC clusters representing an array of different lymphoid, tumor, and stromal cell populations. Multiple other pre-QC clusters exhibited a similar pattern. These data show that abundant imaging artifacts in the TOPACIO dataset not only resulted in an unrealistically large number of clusters, but that these clusters were still under-clustered insofar as they contained cells of different type. We found that redacted cells from both the CRC and TOPACIO datasets showed no discernable pattern in their location, suggesting minimal sampling bias in the QC of these two datasets (Extended data Fig. 6c,d).

Despite significant improvement in both post-QC CRC and TOPACIO clustering, visual inspection revealed clusters with unexpected immunomarker expression patterns. For example, cells in post-QC CRC cluster 13 had high levels tumor/epithelial markers such as Keratin, ECAD, and PCNA, as well as intermediate levels of T cell markers such as CD3, CD45RO, CD45, and CD8α (Extended data Fig. 6e). There is no known cell type that expresses these marker combinations and we found that cluster 13 contained keratin^+^ tumor cells neighboring CD8α^+^ T cells (Extended data Fig. 6f) resulting in some pixels being incorrectly assigned to neighboring cells, a phenomenon referred to as lateral spillover^44^. Future integration of tools for correcting for lateral spillover such as (REDSEA)^44^ into CyLinter may help with this problem, but until then, these instances must be identified by visual inspection.

## DISCUSSION

In this paper we show that artifacts commonly present in highly multiplexed tissue images can have a dramatic impact on single-cell analysis. Artifacts such as tissue folds, lint or hair, antibody aggregates etc. are often outliers with respect to intensity and/or shape and give rise to elements in spatial feature tables that substantially interfere with clustering algorithms such as HDBSCAN, lead to uninterpretable elements in UMAP embeddings, and obscure accurate cell type assignment. Inspection of CyCIF, CODEX, and mIHC images suggests that artifacts can be subdivided into: 1) those intrinsic to the specimen itself (e.g. tissue folds), 2) those arising during staining and image acquisition (e.g. antibody aggregates), and 3) those arising during image-processing (e.g. segmentation errors). The first class is unavoidable and does not usually interfere with visual review by human experts because humans can easily discern and ignore these types of artifacts, but it can dramatically affect computational analysis, particularly of large whole-slide images. The second and third classes of artifacts can be minimized by careful experimental practices and good instrumentation, and we find that as few as 5-10% of cells need to be removed in the best cases. However, specimens that have been mounted on slides many years prior to imaging and stored under suboptimal conditions are more problematic. Unfortunately, specimens of this type are commonly encountered in correlative studies of clinical trial data and high demand for trial specimens means that only one slide is often available for each specimen so it is impossible to go back and fix errors that may arise. In these situations, it is imperative that robust artifact and QC procedures be used so that results can be obtained from invaluable clinical specimens.

Quality control is recognized as a critical step in the acquisition of single cell scRNA-Seq data, and a robust ecosystem of QC tools has been developed in that domain^45,46^. CyLinter is among the first tools to be developed for QC of single-cell data from highly multiplexed tissue images. It works with any TIFF/OME-TIFF files and their corresponding spatial feature tables (CSV format) and is available both as a stand-alone plugin for the popular Napari multi-channel image viewer^25^ and as a component of the MCMICRO^19^ pipeline. CyLinter relies on human visual review, making it effective with a wide range of image and specimen types. However, this process can be time consuming. To make the QC task easier, CyLinter includes a variety of computational approaches for efficiently identifying artifacts or, in some cases, selecting only artifact-free regions of tissue. Currently, CyLinter QC of a ~20-specimen dataset by a single reviewer can take a few days; this compares favorably with several weeks to collect data, and a month or more to perform detailed spatial analysis. In the future, many of these tasks will likely first be performed by machine learning models trained to identify different classes of artifacts. However, we have found that training such classifiers is quite difficult and unsupervised methods have failed thus far. In contrast, many classical image processing approaches – gating for example – have proven effective. Our long-term plan is to incorporate semi-automated machine learning into the human-in-the-loop CyLinter pipeline with a particular focus on automated QC for large batches of similar specimens. It may also be possible to correct for some errors and impute missing values rather than simply redacting data points – the current approach is intentionally conservative in this regard.

When datapoints are removed from a dataset there is always concern that key findings will be biased. This is particularly true in cases such as the TOPACIO dataset in which the majority of the cells were redacted. The same problem holds for scRNA-Seq, although much of the problem arises prior to sequencing, for example during tissue dissociation, microfluidic or flow cytometry sorting, and library preparation^45,47^. In the case of tissue imaging, it is possible to inspect redacted datapoints for patterns. Analysis can then be conditioned on any biases identified. In the case of clinical cohorts, it is particularly important to ensure that that data redaction during QC does not affect one subgroup or trial more than another.

Our experience with over 1,000 whole-slide images from dozens of tissue and tumor types, has continued to demonstrate the necessity of ongoing visual review of high-plex tissue images, ideally with assistance from a trained pathologist. Any hypothesis generated through analysis of data in a spatial feature table must be confirmed through careful inspection of the underlying images, much as sequence polymorphisms were historically validated by review and presentation of the primary data (i.e., gels, sequencer output, etc.). The existing generation of spatial feature tables not only contains errors and omissions, but it also poorly represents much of the morphological information in an image. Continued innovation in QC procedures and image processing algorithms is needed to overcome these issues.

## METHODS

### Software Implementation

CyLinter software is written in Python3, archived on the Anaconda package repository, versioned controlled on Git/GitHub (https://github.com/labsyspharm/cylinter), instantiated as a configurable Python Class object, and validated for Mac and PC operating systems. The tool can be installed at the command line using the Anaconda package installer (see the CyLinter website: https://labsyspharm.github.io/cylinter/ for details) and is executed with the following command: cylinter *configuration.yml*, where configuration.yml is an experiment-specific YAML configuration file. An optional --*module* flag can be passed before specifying the path to the configuration file to begin the pipeline at a specified module. More details on configuration settings can be found at the CyLinter website and GitHub repository (https://github.com/labsyspharm/cylinter^49^). The tool uses the Napari image viewer^50^ for image browsing and annotation tasks. The tool also uses numerical and image-processing routines from multiple Python data science libraries, including pandas, numpy, matplotlib, seaborn, SciPy, scikit-learn, and scikit-image. OME-TIFF files are read using tifffile and processed into multi-resolution pyramids using a combination of Zarr and dask routines that allow for rapid panning and zooming of large (hundreds of GB) images. The CyLinter pipeline consists of multiple QC modules, each implemented as a Python function, that perform different visualization, data filtration, or analysis tasks. Several modules return redacted versions of the input spatial feature table, while others perform analysis tasks such as cell clustering. CyLinter is freely-available for academic re-use under the MIT license. A minimal example dataset consisting of 4 tissue cores from the EMIT TMA22 dataset can be downloaded from the Synapse data repository (Synapse ID: syn52468155) by following instructions at the CyLinter website (https://labsyspharm.github.io/cylinter/exemplar/). All CyLinter analyses presented in this work were performed on a commercially available 2019 MacBook Pro equipped with eight 2.4 GHz Intel Core i9 processors (5.0GHz Turbo Boost) and 32GB 2400MHz DDR4 memory. Imaging data analyzed in this study were stored on and accessed from an external hard drive with 12TB capacity. Implemented software versions are as follows: Python 3.11.5, CyLinter 0.0.47.

### Automated Artifact Detection in CyLinter

An algorithm consisting of classical image analysis steps was designed to automatically identify prevalent artifacts commonly found in highly multiplexed images (e.g., illumination aberrations, antibody aggregates, and tissue folding). The model is applied on a channel-by-channel basis and works on down-sampled versions of each channel, rescaling pixel values to uint8 bit depth for efficient processing. A series of operations in mathematical morphology consisting of erosion and local mean smoothing followed by dilation are applied to transform each down-sampled image channel. These three steps utilize a disk kernel, where the kernel size is a user-defined parameter assumed to have a diameter on the order of 3-5 single cells, conditional on image pixel size. This kernel is then expanded to find local maxima seed points corresponding to putative artifacts. Each artifact is extracted via a flood fill operation according to a specific tolerance parameter that is adjusted in real-time by the user. The union of the flood fill regions produces a binary artifact mask that is resized to the original image dimensions; cells falling within mask boundaries are then dropped from the corresponding spatial feature table.

### t-CyCIF

The CyCIF approach to multiplex imaging involves iterative cycles of antibody incubation with tissue, imaging, and fluorophore deactivation as described previously^26^; protocols and methods related to CyCIF are available on Protocols.io (see “Detailed Experimental Protocols” below). Briefly, multiplex CyCIF images were collected using a RareCyte CyteFinder II HT Instrument equipped with a 20x (0.75 NA) objective and 2x2 pixel binning. This setup allowed for the acquisition of 4-channel image tiles with dimensions 1280x1080 pixels and a corresponding pixel size of 0.65 μm/pixel. All four channels are imaged during each round of CyCIF, one of which is always reserved for nuclear counterstain (Hoechst or DAPI) to visualize cell nuclei. RCPNL files containing 16-bit imaging data were generated (one per image tile) during each imaging cycle.

### Image Processing

Raw microscopy image tiles (RCPNL files) for the datasets described in this study were processed into stitched, registered, and segmented OME-TIFF^51^ files using the MCMICRO image-processing pipeline^29^. Corresponding cell x feature CSV files (i.e., spatial feature tables) were also generated by MCMICRO. Specific algorithms implemented in MCMICRO for the processing of each dataset are as follows: BaSiC—a Fiji/ImageJ plugin for background and shading correction used to perform flatfield and darkfield image correction^52^; ASHLAR—a program for seamless mosaic image processing across imaging cycles^40^; Coreograph (used for the EMIT dataset, https://github.com/HMS-IDAC/UNetCoreograph)—for dearraying the mosaic TMA image into individual TIFF and CSV files per core; UnMICST^41^—used for cell segmentation; employs the U-Net^53^ deep learning architecture for semantic segmentation; S3segmenter (https://github.com/HMS-IDAC/S3segmenter); MCQuant (https://github.com/labsyspharm/quantification) for per cell feature extraction including X,Y spatial coordinates, segmentation areas, mean marker intensities, and nuclear morphology attributes.

### TOPACIO

The TOPACIO dataset used in this study consists of 25 de-identified formalin-fixed, paraffin embedded (FFPE) tissue sections (5 μm thick) of triple-negative breast cancer from patients enrolled in the TOPACIO clinical trial (ClinicalTrials.gov Identifier: NCT02657889). Specimens were collected via one of three different biopsy methods: fine needle, punch needle, or gross tumor resection and procured from Tesaro and Merck pharmaceutical companies as part of the recently-completed trial. Slides were mounted onto Superfrost Plus glass microscope slides (Fisher Scientific, 12-550-15) then dewaxed and antigen-retrieved using a Leica BOND RX Fully Automated Research Stainer prior to multiplex data acquisition by CyCIF. Images were acquired at 20x magnification with 2x2 binning over 10 CyCIF cycles using 27 markers (19 plus Hoechst evaluated in this study).

### CRC

The CRC dataset consists of a whole-slide section (1.6cm^2^) of human colorectal adenocarcinoma tissue (section# 097) imaged at 20x magnification with 2x2 binning over 10 CyCIF cycles using 24 markers across 10 CyCIF cycles (21 plus Hoechst evaluated in the current study) collected as part of the Human Tumor Atlas Network (HTAN).

### EMIT TMA22

The EMIT TMA dataset consists of human tissue specimens from 42 patients organized as a multi-tissue microarray (HTMA427) under an excess tissue protocol (clinical discards) approved by the IRB at Brigham and Women's Hospital (BWH IRB 2018P001627). Two (2) 1.5 mm diameter cores were acquired from each of 60 tissue regions with the goal of acquiring one or two examples of as many tumors as possible (with matched normal tissue from the same resection when feasible). Overall, the TMA contains 123 cores including 3 “marker cores” consisting of normal kidney cortex which were added to the TMA in an arrangement that makes it possible to orient the overall TMA image. Not including the marker cores 44 cores were from males and 76 were from females between 21 and 86 years-of-age. The EMIT TMA22 dataset was acquired at 20x magnification with 2x2 binning over 10 CyCIF cycles using 27 markers (20 plus Hoechst evaluated in the current study) and is available for download from the Synapse data repository (https://www.synapse.org/#!Synapse:syn22345750).

### HNSCC (CODEX)

The HNSCC CODEX dataset consists of two sections of the same deidentified specimen of head & neck squamous carcinoma (HNSCC) imaged at 20x magnification with 2x2 binning over 9 CODEX cycles using 15 markers plus DAPI.

### Normal Tonsil (mIHC)

The mIHC dataset consists of a deidentified whole-slide tonsil specimen from a 4-year-old female of European ancestry procured from the Cooperative Human Tissue Network (CHTN), Western Division, as part of the HTAN SARDANA Trans-Network Project and imaged at 20x magnification with 2x2 binning (0.5 μm/pixel) over 5 mIHC cycles using 18 markers plus Hoechst.

### Detailed Experimental Protocols

1. FFPE Tissue Pre-treatmet Before t-CyCIF on Leica Bond RX V.2 (dx.doi.org/10.17504/protocols.io.bji2kkge)

2. Tissue Cyclic Immunofluorescence (t-CyCIF) V.2 (dx.doi.org/10.17504/protocols.io.bjiukkew)

### Ethics and IRB Statement

The research described in this manuscript was performed on previously published imaging data in part obtained through the Human Tumor Atlas Network (HTAN) and tissue samples from the recently completed TOPACIO clinical trial (ClinicalTrials.gov Identifier: NCT02657889) which was conducted in accordance with ethical principles founded in the Declaration of Helsinki. This study received central approval by the Dana-Farber Cancer Institute (DFCI) institutional review board, protocol 15-550, and/or relevant competent authorities at each clinical trial site. All patients provided written informed consent to participate in the study. All samples and data have been deidentified for the work performed at Harvard Medical School, approved under Institutional Review Boards (IRB) protocol 19-0186. The research complies with all relevant ethical regulations, was reviewed and approved by the IRBs at HMS and DFCI and is considered Non-Human Subjects Research.

## Extended Data

## Supplementary Material

Supplement 1

Supplement 2

## Data Availability

New data associated with this paper is available at the HTAN Data Portal (https://data.humantumoratlas.org). Previously published data is through public repositories. See Supplementary Table 1 for a complete list of datasets and their associated identifiers and repositories. **Online Supplementary Figures 1-4** and the CyLinter demonstration dataset can be accessed at Sage Synapse (https://www.synapse.org/#!Synapse:syn24193163/files)

## Code Availability Statement

CyLinter source code is available for academic re-use under the MIT open-source license agreement at Github (https://github.com/labsyspharm/cylinter)^49^. Code used to produce the findings of the study is available at https://github.com/labsyspharm/cylinter-paper.
